# Skin temperature changes after ultrasound-guided supra-inguinal fascia iliaca block: a prospective observational study

**DOI:** 10.1186/s40981-021-00435-x

**Published:** 2021-04-05

**Authors:** Manabu Yoshimura, Hiroko Shiramoto, Mami Koga, Aya Yoshimatsu, Yasuhiro Morimoto

**Affiliations:** Department of Anesthesiology, Ube Industries Central Hospital, 750 Nishikiwa, Ube City, Yamaguchi, 755-0151 Japan

**Keywords:** Supra-inguinal fascia iliaca block, Skin temperature, Thermography

## Abstract

**Purpose:**

Ultrasound-guided supra-inguinal fascia iliaca block (SFIB) is widely used as regional anesthesia of the hip and thigh. It is difficult to judge the blocking effect and the spreading local anesthesia. We hypothesize that the effect and spread of the block could be proven objectively by a rise in the temperature. In this prospective observational study, the broad regional rise in skin temperature of twenty patients who were scheduled for hip surgery was measured using an infrared thermographic camera at multiple intervals following ultrasound-guided SFIB.

**Methods:**

Infrared thermographic imaging of skin temperature at the femoral, obturator, and lateral femoral cutaneous nerve sites was performed before and at 5-min intervals after ultrasound-guided SFIB for up to 15-min post-injection. The primary outcomes are skin surface temperature. Sensory block was assessed immediately after the final infrared thermographic image acquisition using the cold test.

**Results:**

Compared to pre-injection baseline, temperature increased by 1.2 °C [95% confidence interval (CI) 0.4–2.0 °C] after 5 min, 1.2 °C (95% CI 0.4–2.0 °C) after 10 min, and 0.9 °C (95% CI 0.4–2.1°C) after 15 min. The cold test response was reduced in all cases at the femoral and lateral femoral cutaneous nerve sites and in 13 cases at the obturator nerve site. The sensitivity and specificity of the temperature increase to cold loss were 96% and 63%, respectively when we defined >0°C as the clinical threshold.

**Conclusions:**

Successful SFIB significantly enhanced skin temperature at the hip and thigh in all cases, suggesting that infrared surface thermography can be used as an objective assessment tool for adequate analgesia.

**Trial registration:**

University Hospital Medical Information Network Clinical Trials Registry (UMIN 000037866). Registered 31 August 2019.

## Introduction

Regional anesthesia has a positive impact in the treatment of postoperative pain after hip surgery. Recently, ultrasound-guided supra-inguinal fascia iliaca block (SFIB) has been widely used as the regional anesthesia of the hip and thigh [1–3]. The fascia iliaca is a connective tissue layer on the surface of the iliac and psoas muscles. The virtual space between fascia iliaca and muscles covered by the fascia forms the fascia iliaca compartment. The primary nerves of the lumbar plexus, the femoral, obturator, and lateral femoral cutaneous nerves (LFCN), are contained within the fascia iliaca compartment as they travel on the iliacus muscle and caudally between iliacus and psoas muscles. A high-dose injection of local anesthesia is successful if the cranial spread of the local anesthetic is present under the fascia iliaca.

It is difficult to objectively judge the blockade of the femoral, obturator, and LFCN, and the spread of local anesthetics, which is the same as fascial plane block in general. The measurement of skin temperature has been useful in determining the blocking effect in the sciatic nerve and brachial plexus blocks [4–7]. To our knowledge, no study has reported SFIB along with measurement of the skin temperature. A number of studies have shown that increased skin temperature after blocking is closely associated with block effect and spread [5, 7–9]. We hypothesized that the effect and spread of the block (femoral, obturator, and LFCN) could be proven objectively by a rise in the temperature. Therefore, we used an infrared thermographic camera to measure the broad regional increase in skin temperature at multiple time intervals following ultrasound-guided SFIB.

## Methods

This prospective, single-arm, observational study is reported according to the Strengthening the Reporting of Observational Studies in Epidemiology (STROBE) consensus guidelines. This study was approved by the Institutional Review Board (2019-0532: Ube Industries Central Hospital, Japan) and registered in the University Hospital Medical Information Network Clinical Trials Registry (UMIN 000037866). Written informed consent was obtained from all patients.

We selected patients from 31 August 2019 to 26 November 2019, who were scheduled for hip surgery and had chosen ultrasound-guided SFIB followed by general anesthesia. Exclusion criteria were age <20 years, peripheral neurological or vascular diseases, general contraindications to local anesthesia (e.g., increased risk of bleeding, infection at the injection site, allergy to local anesthetics), inability to communicate (e.g., due to dementia or other neuropsychiatric diseases), and refusal to participate.

### Ultrasound-guided supra-inguinal fascia iliaca block

Ultrasound-guided SFIB was performed as described by Hebbard et al. [10] A liner ultrasound probe (HFL38 6–13 MHz, S-nerve ultrasound System, Sonosite Inc., Bothell, WA, USA) was placed in the sagittal plane to obtain an image of the anterior superior iliac spine. The fascia iliaca and sartorius, iliopsoas, and oblique internal muscles were identified by sliding the probe medially. After identifying the bow-tie sign formed by the muscle fasciae, a 100-mm 20G nerve block needle (UNIEVER DISPOSABLE NERVE BLOCKADE NEEDLE WITH HUBER POINT, UNISIS Corp., Saitama Japan) was introduced 1-cm cephalad to the inguinal ligament (IL). Using an in-plane approach, the fascia iliaca was penetrated and separated from the iliac muscle by hydrodissection. Within the space created by hydrodissection, the needle was advanced further in a cranial and slightly dorsal direction. Upward movement of the overlying deep circumflex artery upon injection was used as a sign of successful fascia iliaca penetration. A total volume of 30 ml levobupivacaine 0.25% was injected. Injection was considered successful if spread of local anesthesia was observed cranial to the point where the iliac muscle passes under the abdominal muscles. If spread was insufficient, the injection was stopped and the needle repositioned until adequate spread was obtained. All SFIB procedures were performed by the same experienced anesthesiologist (M.Y).

General anesthesia was administered after assessing the temperature. A supraglottic airway was inserted after the induction of anesthesia with sevoflurane, remifentanil, and rocuronium. Anesthesia was maintained with sevoflurane and remifentanil. Fentanyl was used in the range of 0–2 μg/kg.

### Block assessment

To avoid interference with skin temperature measurements, sensory block was assessed immediately following the final infrared thermographic image acquisition using the cold test. Tests were conducted in succession at the femoral, obturator, and LFCN sites.

### Infrared thermographic imaging

Infrared thermographic images were acquired using a FLIR ONE infrared camera (FLIR Systems, Wilsonville, US) with 0.1°C resolution. During measurements, the camera was set according to the actual distance from the patient (1.0 m), room temperature (20.0°C), air humidity, and the emission coefficient for the human body (0.9). Three pictures were taken from the lateral thigh, thigh, and medial bottom side of the hip perpendicular to the surface at distance of 0.8 m. No interventions were performed during infrared imaging, and the patients were instructed not to move their legs during the measurement period.

### Temperature assessment

Primary outcome was skin temperature after block compared before block. Infrared thermographic images of the hip and thigh were acquired before and at 5-min intervals after ultrasound-guided SFIB for up to 15-min post-injection. A preliminary assessment of skin temperature distribution was conducted in the operating room, and then, absolute values were derived after surgery at femoral, obturator, and LFCN sites (measurement area of 150 × 100 pixels) using a computer software (FLIR Systems, Wilsonville, OR, USA). Heat maps were created for each site with a temperature increase of >0°C defined as the success between baseline and 5, 10, or 15 min.

### Statistical analysis

We used data from a past study by Park et al. [11] to estimate the sample size needed to detect a significant difference in skin temperature. Assuming a mean change in skin temperature of 2.0°C and standard deviation of 0.8, 20 patients would be required for 80% statistical power (beta) at an alpha level (two-sided) of 5%. All statistical analyses were conducted using SPSS version 24 for Windows (SPSS, Chicago, IL, USA).

We analyzed differences among measurement times and sites using linear mixed-effects models accounting for repeated measures. Least square means and 95% confidence intervals (CI) are reported for these models. Bonferroni contrasts were calculated for comparison among measurement times. Data are expressed as mean ± standard deviation. A *p* <0.05 was considered statistically significant for all tests.

Results

**Fig. 1:**
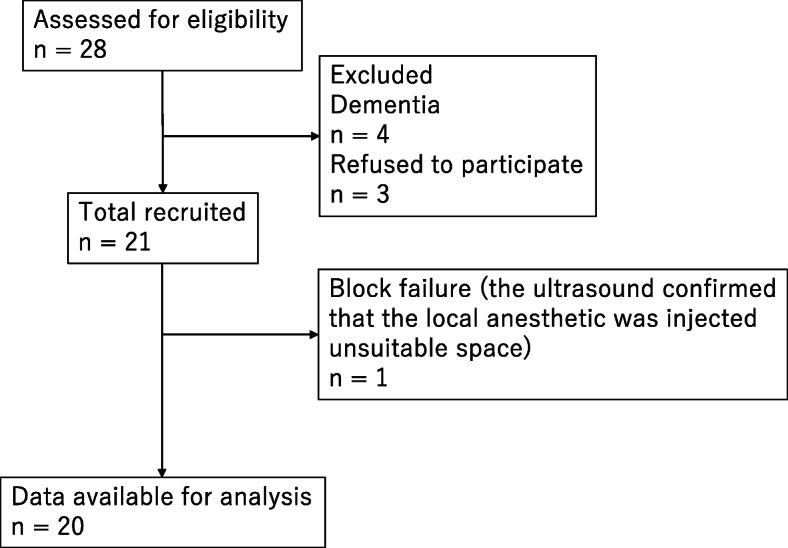
Flow diagram of patient selection

**Fig. 2:**
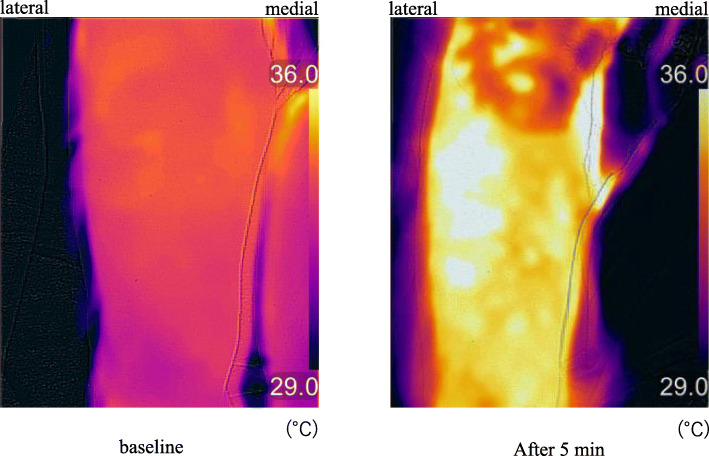
Thermographic images acquired before and 5 min after ultrasound-guided supra-inguinal fascia iliaca block. Thermography predominantly in the lateral femoral nerve site in patient no. 1 resulted in an increase in skin temperature in the middle of lateral femoral site was increased from 33.6 to 35.8°C by nerve block

**Fig. 3:**
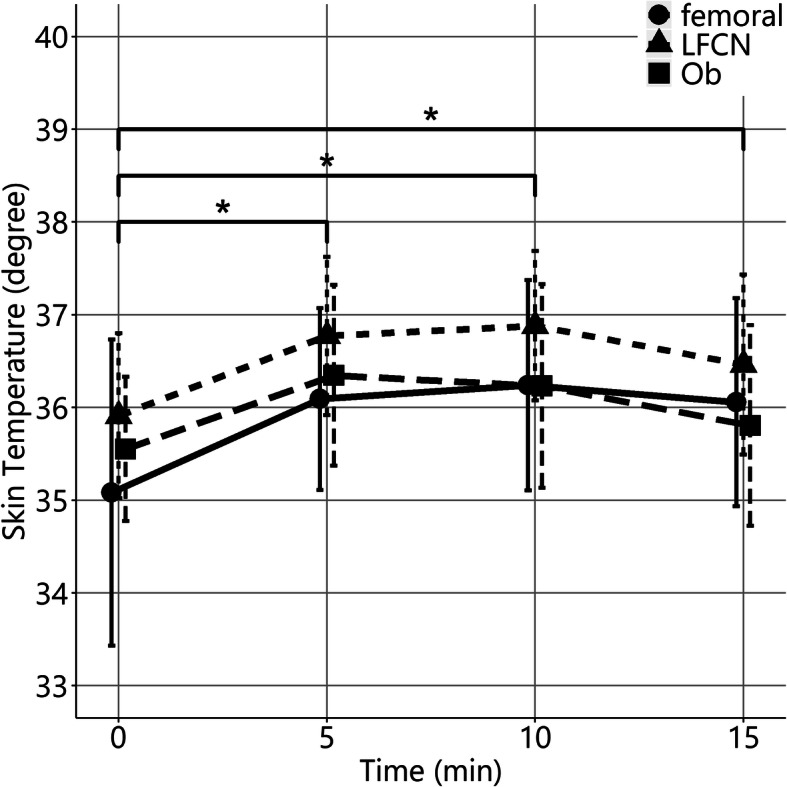
Skin temperature at the femoral nerve, lateral femoral cutaneous nerve, and obturator nerve sites at baseline and at 5 min intervals after ultrasound-guided SFIB. Data are expressed as mean ± standard deviation of 20 experiments. *P < 0.01 compared with baseline including all data of the femoral, lateral femoral cutaneous, and obturator nerves site from 20 patients. LFCN, lateral femoral cutaneous nerve site; Ob, obturator nerve site

**Fig. 4:**
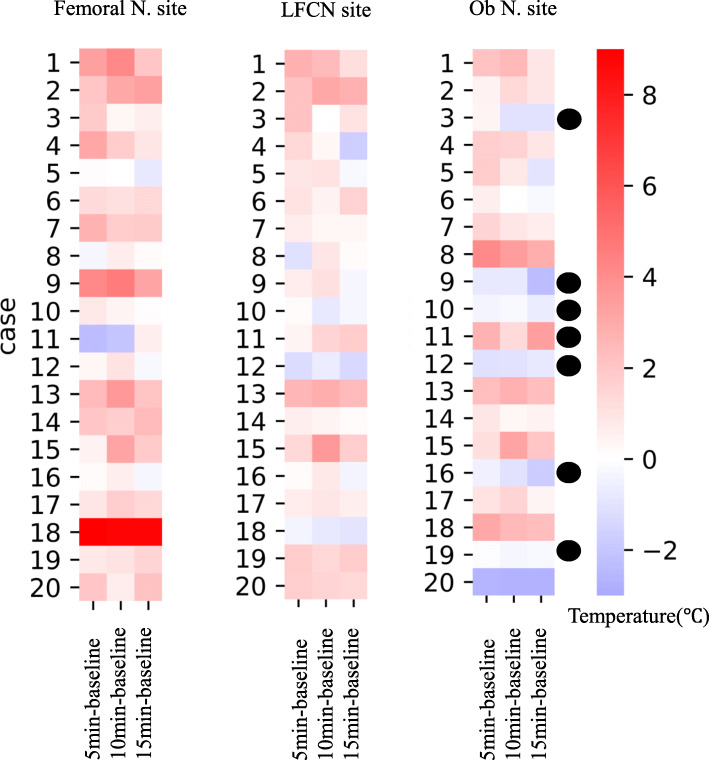
Heat maps for each site. A temperature increase of >0°C was defined as the success between baseline and 5, 10, or 15 min. The sensitivity and specificity of the temperature increase to the cold loss were 96% and 63%, respectively. Black circle, no cold loss; LFCN, lateral femoral cutaneous nerve; Ob, obturator nerve

Twenty-eight consecutive patients were deemed eligible during the study period from August to November 2019, of which four patients were excluded due to dementia and three refused to participate (Fig. 1), and the remaining 20 patients (2 males and 18 females, aged 82 ± 4 years) were included. The number of patients with ASA physical status 1, 2, and 3 was 1, 4, and 15, respectively. Thermographic images are shown in Fig. 2. The temperature mean increased from 35.5 to 36.7 °C after 5 min, to 36.7°C after 10 min, and to 36.4°C after 15 min. The mean temperature including the skin area of the three nerves 5, 10, and 15 min after nerve block was significantly increased compared with the baseline (*P* < 0.01 for all, Fig. 3). The cold test response was reduced in all cases at the femoral and LFCN sites and in 13 cases at the obturator nerve site. Figure 4 displays heat maps for each site with a temperature >0°C defined as the success between baseline and 5, 10, or 15 min. The sensitivity and specificity of the temperature increase to the cold loss were 96% and 63%, respectively when we defined >0°C as the clinical threshold.

## Discussion

We conducted a prospective observational study for the changes in skin temperature following ultrasound-guided SFIB in 20 patients scheduled for hip surgery. We found that temperature significantly increased overall during the reduced cold test.

Recently, “supra-inguinal” fascia iliaca block has been proven to be more effective than mere fascia iliaca block [1], especially the longitudinal approach of SFIB that enhances the expression of local anesthetics in the central part of the body. Therefore, the SFIB longitudinal approach can block the femoral and LFCN at a high rate. In addition, it is highly likely that the obturator nerve will be blocked, which is similar to a lumbar plexus block.

The main purpose of the present study was to characterize the surface thermographic responses of the hip and thigh after ultrasound-guided SFIB. Infrared imaging revealed a significant proximal gradient in baseline temperatures and a significant rise at the hip and thigh following ultrasound-guided SFIB. There was also a strong association between cold testing response and skin temperature rise, suggesting that surface infrared thermography may be a feasible tool for non-invasive evaluation of block success, especially in patients with contraindications to other assessment measures.

Our study indicated that SFIB may block the femoral, obturator, and LFCN, as shown in cadaver studies [10] and our study in a clinical setting. Thermography can be useful in confirming blocking effects clinically.

No previous studies have investigated the infrared thermographic response after specific peripheral nerve block, and only a few have investigated temperature changes after hip and thigh block [9]. Stevens et al. [5] reported that skin temperature changes at the medial ankle and medial mid-tibia were minimal and developed slowly following femoral nerve block, whereas such changes were undetectable at the hip and thigh. In contrast, blockade of the femoral, obturator, and LFCN did appear to induce reliable changes in hip and thigh skin temperatures that were strongly associated with block success as confirmed by the cold test response.

Several previous studies have also demonstrated a relationship between successful nerve block and thermographic response in other regions [4]. For instance, Stevens et al. [5] reported a skin temperature increase of 1.8°C ± 0.9°C at the foot 5 min following sciatic nerve block. Several studies have also shown that the skin temperature increases in the upper extremity after brachial-specific nerve block. Lange et al. [7] reported that specific ulnar and median nerve block markedly increased mean skin temperatures of the innervated areas (5.2°C and 5.1°C, respectively), with even larger increases at the fingertips. Similarly, Asghar et al. [6] reported a significant increase (6.6°C) on the medial-distal skin of the thumb 5 min following interscalene brachial plexus block. The larger changes at the extremities compared to the trunk may be explained by opening of arteriovenous anastomoses caused by blockade of specific sympathetic nerve fibers.

A critical determinant of SFIB success is the local spread of anesthetic. There are no detailed studies regarding how much local anesthesia is required with SFIB. This study objectively demonstrated that the percentage of obturator nerve block using 30 ml of local anesthesia was 65%, in accordance with a previous cadaver study [10]. Therefore, our results may be broadly applicable to cases using the SFIB method of Hebbard et al. [10]. The crucial difference between conventional temperature studies of lower extremity blocks and our study is the observation of drug spreading. It could be objectively proven using thermography that LFCN was blocked. We were able to objectively prove the blocking effect in vivo.

Limitations of the present study include the relatively short postoperative thermographic measurement period (15 min) compared to some previous studies due to intraoperative time constraints. Nonetheless, our results indicate that thermographic measurements can permit rapid evaluation of block success, consistent with study aims. Second, we were unable to measure the temperature of the contralateral lower extremity because we considered it unethical to spend a long time taking images of the lower body of a conscious patient with a camera. Finally, the innervation area of the obturator nerve is uncertain and requires further study for more precise characterisation of analgesic spread.

## Conclusion

Skin temperature was significantly higher at 5 min, 10 min, and 15 min following SFIB than that at pre-injection baseline. Infrared surface thermography can be used as an objective assessment tool for adequate analgesia.

## Data Availability

The datasets analyzed during this study are available from the corresponding author on reasonable request.

## References

[1] Desmet M, Vermeylen K, Van Herreweghe I, Carlier L, Soetens F, Lambrecht S (2017). A longitudinal supra-inguinal fascia iliaca compartment block reduces morphine consumption after total hip arthroplasty. Reg Anesth Pain Med.

[2] Gasanova I, Alexander JC, Estrera K, Wells J, Sunna M, Minhajuddin A, Joshi GP (2019). Ultrasound-guided suprainguinal fascia iliaca compartment block versus periarticular infiltration for pain management after total hip arthroplasty: a randomized controlled trial. Reg Anesth Pain Med.

[3] Steenberg J, Moller AM (2018). Systematic review of the effects of fascia iliaca compartment block on hip fracture patients before operation. Br J Anaesth.

[4] Hermanns H, Werdehausen R, Hollmann MW, Stevens MF (2018). Assessment of skin temperature during regional anaesthesia-what the anaesthesiologist should know. Acta Anaesthesiol Scand.

[5] Stevens MF, Werdehausen R, Hermanns H, Lipfert P (2006). Skin temperature during regional anesthesia of the lower extremity. Anesth Analg.

[6] Asghar S, Bjerregaard LS, Lundstrom LH, Lund J, Jenstrup MT, Lange KH (2014). Distal infrared thermography and skin temperature after ultrasound-guided interscalene brachial plexus block: a prospective observational study. Eur J Anaesthesiol.

[7] Lange KH, Jansen T, Asghar S, Kristensen PL, Skjonnemand M, Norgaard P (2011). Skin temperature measured by infrared thermography after specific ultrasound-guided blocking of the musculocutaneous, radial, ulnar, and median nerves in the upper extremity. Br J Anaesth.

[8] van Haren FG, Driessen JJ, Kadic L, van Egmond J, Booij LH, Scheffer GJ (2010). The relation between skin temperature increase and sensory block height in spinal anaesthesia using infrared thermography. Acta Anaesthesiol Scand.

[9] Werdehausen R, Braun S, Hermanns H, Freynhagen R, Lipfert P, Stevens MF (2007). Uniform distribution of skin-temperature increase after different regional-anesthesia techniques of the lower extremity. Reg Anesth Pain Med.

[10] Hebbard P, Ivanusic J, Sha S (2011). Ultrasound-guided supra-inguinal fascia iliaca block: a cadaveric evaluation of a novel approach. Anaesthesia.

[11] Park SY, Nahm FS, Kim YC, Lee SC, Sim SE, Lee SJ (2010). The cut-off rate of skin temperature change to confirm successful lumbar sympathetic block. J Int Med Res.

